# A Lack of Bioactive Predictability for Marker Compounds Commonly Used for Herbal Medicine Standardization

**DOI:** 10.1371/journal.pone.0159857

**Published:** 2016-07-26

**Authors:** Guillermo G. Ruiz, Erik O. Nelson, Adam F. Kozin, Tiffany C. Turner, Robert F. Waters, Jeffrey O. Langland

**Affiliations:** 1 Southwest College of Naturopathic Medicine, Tempe, AZ 85282, United States of America; 2 Arizona State University, Biodesign Institute, Tempe, AZ 85287, United States of America; University of New South Wales, AUSTRALIA

## Abstract

The use of botanical medicine by practitioners and the general public has dramatically increased in recent years. Most of these botanical therapeutics are obtained through commercial manufacturers or nutraceutical companies. The current standard of practice that manufacturers typically use to standardize botanicals is done based on the level of a well-known, abundant marker compound present in the botanical. This study evaluated the putative correlation between the level of a marker compound and the biological activity of eight common botanicals. Overall, the standardization of a botanical based on a marker compound was found not to be a reliable method when compared to *in vitro* bioactivity. A marker compound is often not the biologically active component of a plant and therefore the level of such a marker compound does not necessarily correlate with biological activity or therapeutic efficacy.

## Introduction

In the last three decades, botanical medicine has experienced a substantial growth in popularity. In the United States, approximately 38% of adults and approximately 12% of children are using some form of complimentary and alternative medicine (CAM). Of these, natural products, including botanicals, are the most common form of CAM therapy used [1]. Worldwide, the World Health Organization estimates that 80% of people rely on herbal medicines for some part of their primary health care [2]. In Australia, Canada, USA, Belgium, and France, it is estimated that 48%, 70%, 42%, 38%, and 75% of people, respectively, have used herbal medicines at least once for their healthcare needs [3,4]. Much of the driving force behind this desire for herbal medicine is associated with public dissatisfaction with the cost of prescription medications, concerns over potential side-effects with conventional medications, and a growing interest in natural or organic remedies.

As phytotherapeutics continue to gain popularity in global markets, increased oversight and means of quality control become paramount to ensuring the medicinal value and activity of therapeutic botanicals. Standardization, a concept modelled largely from the pharmaceutical industry involving the identification of an active constituent or marker compound, has been adopted by both nutraceutical companies and government agencies as a means for quality control of phytotherapeutics [5,6,7,8]. This approach seeks to establish the level of a single phytochemical biomarker within a species to hopefully predict for the pharmacological bioactivity of botanical supplements. Although a commonly accepted practice for quality assurance in the nutraceutical market, limited research has evaluated standardization using a single marker compound as an efficacious model of overall product quality in terms of bioactivity and potency.

Unlike a conventional pharmaceutical product which usually contains a single defined chemical, botanical extracts contain mixtures of multiple components. Different preparations for the same species of plant may vary in their chemical profile dependent upon geographical area, seasonal variations, method of harvest, extraction procedures, and/or storage conditions [5,6,8,9]. In addition, a botanical extract may contain numerous active constituents which may act synergistically or target multiple conditions or diseases. Very few botanicals with observed therapeutic effects have well-defined ‘active components’ which have been chemically identified. Due to this, a marker compound, which is a constituent having an established chemical structure, is accepted as the industry standard for quality control of nutraceuticals even if that compound is not directly involved in the biological activity of the herb.

This study sought to explore the relationship or correlation between the standard marker compound from selected botanical species and the predictive efficacy based on *in vitro* bioactivity.

## Materials and Methods

### Botanical selection and preparation

The botanicals selected for this study were selected based on their established antibacterial, antifungal, antiviral or immune-stimulatory activity. Dried plant material was obtained from reputable sources with documentation of authenticity. All plant material was subsequently verified by qualified botanical specialists using herbal pharmacopoeia monographs and reference keys. A voucher specimen of all plant material was deposited in a repository. For tincture preparation, the dried botanicals were ground to a fine powder, resuspended in extraction solution and incubated for 3 days at room temperature. The extract was centrifuged at 3000 x g for 10 min to remove cell debris and the extraction solution filtered through a 0.2 micrometer filter. For standardization and comparison, all botanical extracts had an average non-volatile constituent concentration averaging 38.4 mg/ml (ranging from 28.4–42.6 mg/ml). This concentration (mg/ml) is based on the weight of non-volatile constituents present in the extract per ml of aqueous liquid. The source, extraction solution, and plant solid to liquid ratio (grams:ml) include: *Turnera diffusa* (Starwest botanicals [Sacramento, CA], AmeriHerb [Ames, IA], Mountain Rose [Eugene, OR] 1:4 leaf extract with 63% ethanol, 27% water, 10% glycerin), *Eucalyptus globulus* (Starwest botanicals [Sacramento, CA], AmeriHerb [Ames, IA], Mountain Rose [Eugene, OR] 1:3 leaf extract with 53% ethanol, 42% water, 5% glycerin), *Cinnamomum zeylanicum* (Starwest botanicals [Sacramento, CA], AmeriHerb [Ames, IA], Mountain Rose [Eugene, OR] 1:8 bark extract with 65% ethanol, 35% water); *Piper cubeba* (Starwest botanicals [Sacramento, CA], Dragon herbarium [Ames, IA], Nature’s Wonderland [Eugene, OR] 1:8 berry extract with 60% ethanol, 40% water), *Hypericum perforatum* (Starwest botanicals [Sacramento, CA], AmeriHerb [Ames, IA], Mountain Rose [Eugene, OR] 1:4 leaf extract with 58% ethanol, 32% water, 10% glycerin), *Glycyrrhiza glabra* (Starwest botanicals [Sacramento, CA], AmeriHerb [Ames, IA], Mountain Rose [Eugene, OR] 1:6 root extract with 26% ethanol, 10% glycerin, 64% water), *Echinacea purpurea* (Starwest botanicals [Sacramento, CA], AmeriHerb [Ames, IA], Mountain Rose [Eugene, OR], 1:5 leaf/flower extract with 25% ethanol, 75% water), *Astragalus membranaceus* (Starwest botanicals [Sacramento, CA], AmeriHerb [Ames, IA], Mountain Rose [Eugene, OR] 1:5 root extract with 25% ethanol, 75% water).

Marker compound analysis: The analytical measurement of marker compound levels was done commercially by ChromaDex (Irvine, CA) for each botanical using standard procedures. An Agilent 1100 Series system was used and all samples compared to the known reference standards. Analytes (marker compounds) chosen to be measured were based on established identification and characterization of each marker compound within each herb. An analytical test report was obtained for each sample and the percent value for the desired analyte (marker compound) reported relative to the weight of the entire sample. The analytes for each botanical included: *Eucalytpus globulus* (marker compound: eucalyptol), *Turnera diffusa* (marker compound: arbutin), *Glycyrrhiza glabra* (marker compound: glycyrrhizic acid), *Hypericum perforatum* (marker compound: hyperforin), *Cinnamomum burmanii* (marker compound: coumarin), *Piper cubeba* (marker compound: piperine), *Echinacea purpurea* (marker compounds: caftaric acid, echinacoside and cichoric acid), and *Astragalus membranaceus* (marker compound: astragaloside I).

### Anti-bacterial assay

Media and the bacterial culture *Staphylococcus aureus* ATCC 11632 were obtained from Hardy Diagnostics (Santa Monica, CA). For minimum inhibitory concentration (MIC) determination, 18-hour cultures (ranging from 1-5x10^8^ colony forming units (CFU)/ml) were diluted into media (1:1,000 dilution; tryptic soy broth (TSB)) followed by the addition of varying concentrations of each botanical extract. The cultures were incubated at 37°C with aeration (by continuous rotation) for 24 hours. The MIC value was determined as the dose of the botanical extract required to completely inhibit replication of the bacteria (as measured by a lack of turbidity absorbance.

### Anti-fungal assay

Media and the yeast culture *Candida albicans* ATCC 10231 were obtained from Hardy Diagnostics (Santa Monica, CA). For growth studies, 18-hour cultures (ranging from 3-8x10^7^ colony forming units (CFU)/ml) grown at 30°C in YPD broth were diluted into media (1:1,000 dilution; yeast peptone dextrose broth (YPD with 5% fetal bovine serum)) followed by the addition of varying concentrations of each botanical extract. For hyphal growth assessment, the cultures were incubated 37°C with aeration (by continuous rotation) for 24 hours. At 24 hours post-treatment, light microscopy was done to evaluate the morphological state of the *C*. *albicans*. Each broth culture was stained with Lactophenol Cotton Blue and visualized by light microscopy. A minimum of 100 cells were counted and the percent of single cells verses hyphal/pseudohyphal cells determined.

### Anti-viral assay

HSV1 KOS (a kind gift from David Bloom, Univ. of Florida College of Medicine). Vero cells (ATCC) were maintained with Minimal Essential Media (Cellgro) supplemented with 100 IU penicillin/ml, 100 microgram streptomycin/ml, 2.5 microgram amphotericin B/ml, and 10% heat-inactivated fetal bovine serum (Hyclone). Cells were incubated at 37°C, 5% CO2 in a humidified chamber. Plaque reduction assays were performed by diluting virus stocks and preincubating 100–200 plaque-forming units (pfu) with increasing concentrations of botanicla extract for 20 minutes. Monolayers were infected for 1 hour at 37°C followed by incubation in media containing the botanical for 3 days at 37°C. Plaques were visualized by staining with 0.1% crystal violet in 20% ethanol.

### Immune-stimulatory assay

Human PBMCs were obtained from freshly drawn blood. To obtain the fresh PBMCs, blood was collected by venipuncture into heparinized tubes. Whole blood was removed and added to an equal volume of balanced salt solution (0.01% D-glucose, 0.005 mM CaCl_2_, 0.098 mM MgCl_2_, 0.54 mM KCl, 14.5 mM Tris pH 7.6, 126 mM NaCl). Forty mls of blood/salt solution was layered on top of 10 mls Ficoll-Paque Plus (Amersham Biosciences) and centrifuged at 400×g for 40 minutes at 20°C. PBMCs were removed from the interface and washed in balanced salt solution. The collection of fresh PBMCs was approved by the Arizona State University and Southwest College of Naturopathic Medicine (SCNM) Institutional Review Boards. All participants signed a written informed consent form (approved by the SCNM Institutional Review Board) prior to participation. PBMCs were resuspended in RPMI1640 with 10% fetal bovine serum (1×10^6^ cells/mL) in cell culture dishes. 1×10^7^ cells (in 10 mL media) were treated with botanical extract for 18 hours. Following treatment, total RNA was isolated and purified as per manufacturer’s protocol using an RNeasy kit (Qiagen). Briefly, the cell lysate was homogenized using a QIAshredder spin column. Any DNA contamination was eliminated by DNase digestion and the RNA was isolated using the RNeasy spin column. Quantitative real-time PCR (qPCR) was performed using iQSYBR Green Super mix (Bio-Rad). Briefly, cDNA template for each isolate was added to wells containing PCR reaction mix (iQSYBR Green Super mix and primers). Primers were obtained from SABiosciences. Reactions were done in a MiniOpticon real-time PCR detection system with CFX Manager software control (Bio-Rad). All samples were normalized to GAPDH [10].

### Statistical analysis

SPSS^™^ statistical software was used to analyze the data. Comparing marker compound with biological activity values, including the three (3) “Sources”, a Paired T-test was used. Further analysis was done to determine individual group cause and effect relationships between “Marker Compound” and “Biological Activity”. A regression line comparison (Co-Variance) was computed compared to a “CONTROL” regression line on each of the botanicals. 

## Results

**Fig 1:**
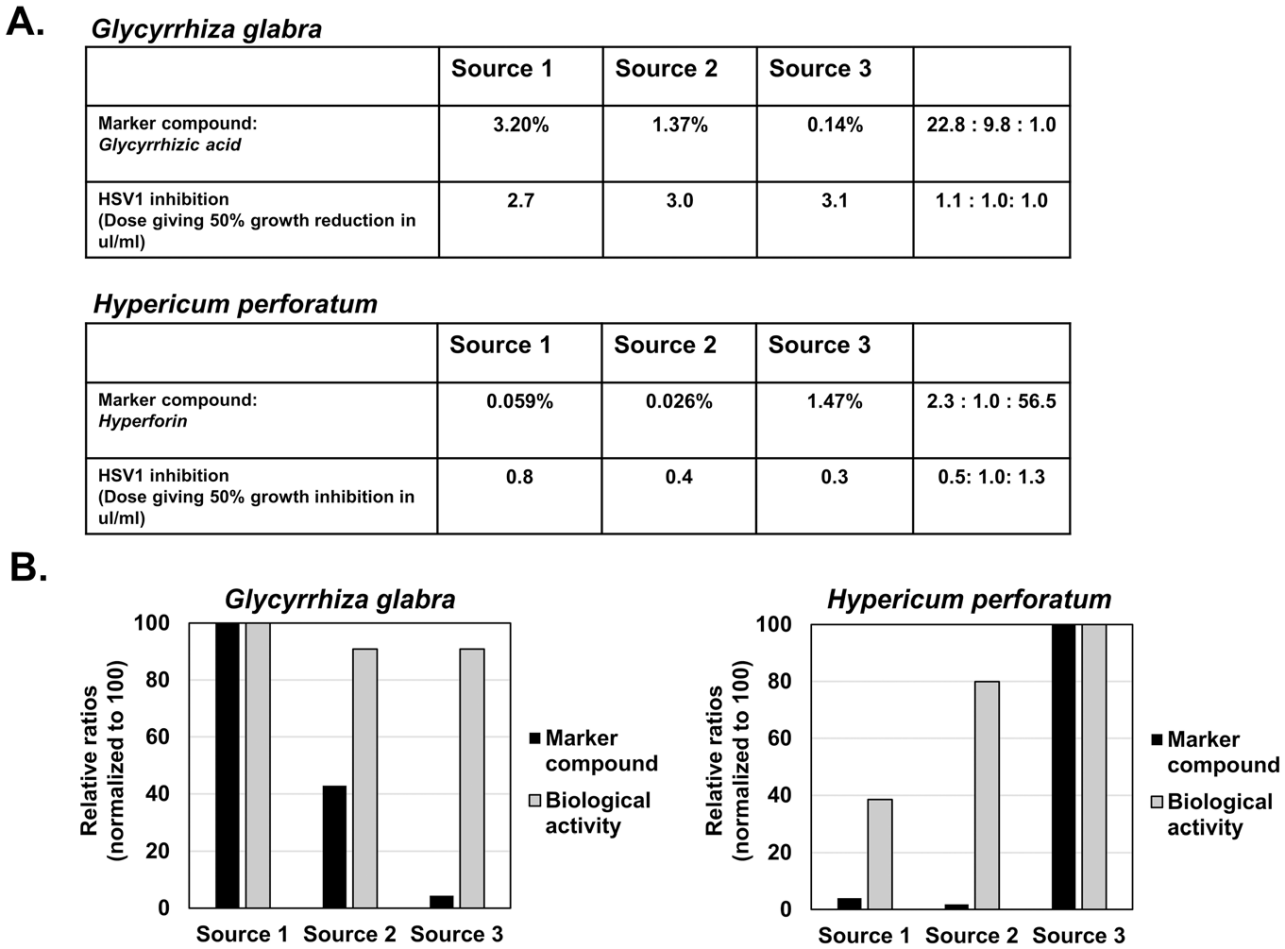
Marker compound level and bioactivity of antiviral herbs. Part A) Marker compound level (percent of total weight) of glycyrrhizic acid (from *G*. *glabra)* and hyperforin (from *H*. *perforatum)* and HSV-1 ID_50_ values (μl/ml) are shown from three commercial sources. Part B) Marker compound ratios and bioactivity ratios were normalized to 100 (max. level) and graphed.

Dried herbal samples were obtained from three different reputable, commercial sources. Each species of herb was extracted under the same conditions. Fig 1 illustrates the marker compound level and *in vitro* bioactivity results for the antiviral herbs, *Glycyrrhiza glabra* and *Hypericum perforatum*. As shown in Fig 1A, the level of the each marker compound varied substantially between the different sources with glycyrrhizic acid at 3.2%, 1.37% and 0.14% of the relative weight of the sample, and hyperforin at 0.059%, 0.026% and 1.47%. Antiviral activity of the extracts was measured as the dose required to inhibit herpes simplex-1 (HSV-1) plaque formation by 50% (ID_50_). For *G*. *glabra*, the ID_50_ was reasonably consistent between all three samples (or commercial sources), even though the level of glychyrrhic acid was highly variable (Fig 1A and 1B). For *H*. *perforatum*, the viral ID_50_ varied between the samples, but did not correlate well with the level of hyperforin (Fig 1A and 1B). These results support that the marker compound for these herbs is not a reliable measure of antiviral activity associated with these herbs and that these individual compounds are not likely involved in this bioactivity.

**Fig 2:**
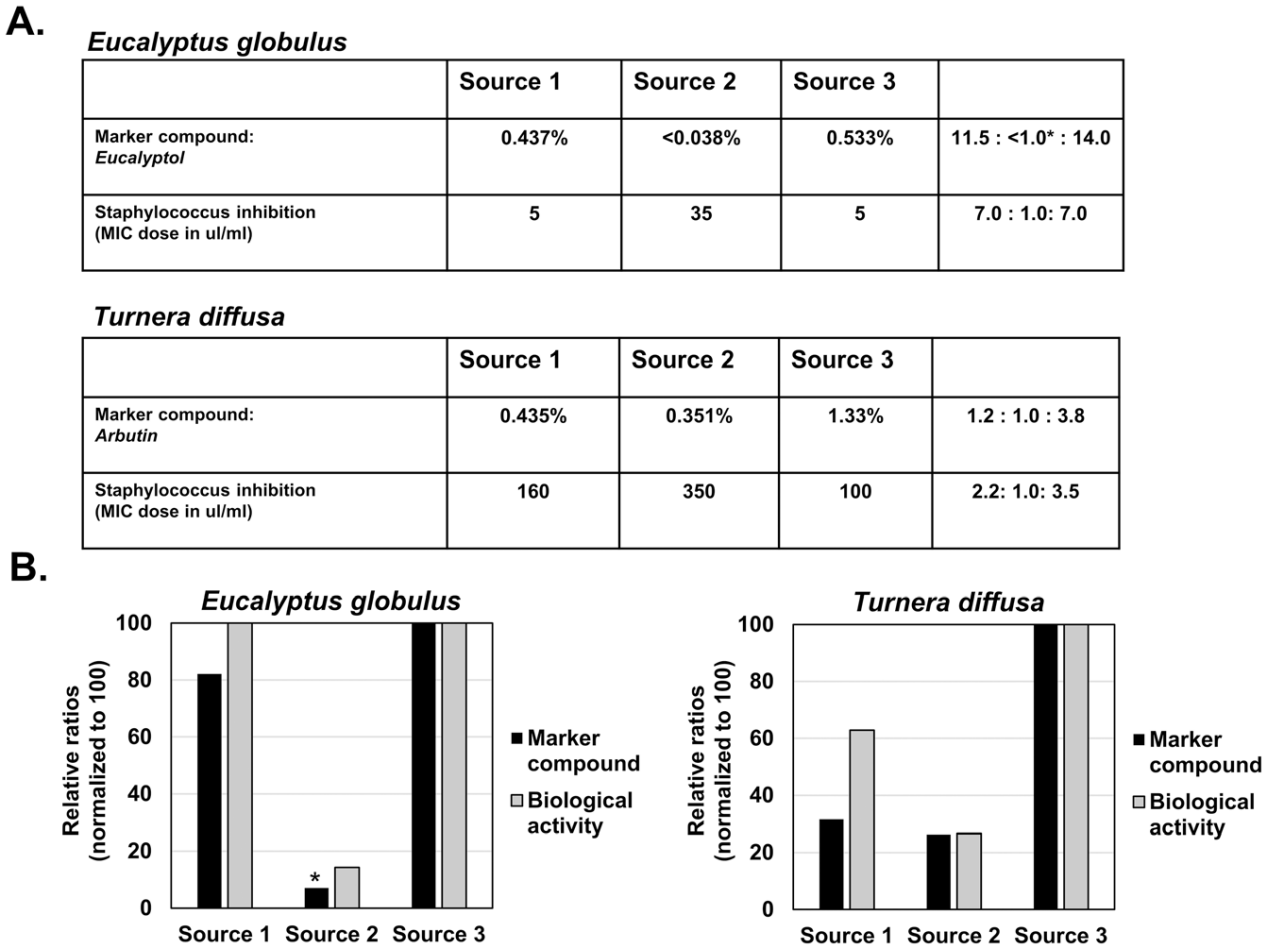
Marker compound level and bioactivity of antibacterial herbs. Part A) Marker compound level (percent of total weight) of eucalyptol (from *E*. *globulus)* and arbutin (from *T*. *diffusa)* and *Staphylococcus aureus* MIC values (microliters/ml) are shown from three commercial sources. Part B) Marker compound ratios and bioactivity ratios were normalized to 100 (max. level) and graphed.

For comparison to antibacterial activity, *Eucalyptus globulus* and *Turnera diffusa* were selected based on their historical use [11,12,13]. As shown in Fig 2A, the levels of eucalyptol in the samples were at 0.437%, <0.038% and 0.533%, and arbutin at 0.435%, 0.351% and 1.33%. Again, these results suggest that significant differences exist between the levels of each marker compound when herbs are obtained from varying sources. Antibacterial activity was measured as the dose of each extract required to obtain the MIC (minimum inhibitory concentration). As shown, eucalyptol appeared to illustrate a positive correlation between the antibacterial activity and the marker compound level (Fig 2B). Similarly, although not as correlative, extracts from *T*. *diffusa* appeared to demonstrate that as changes in arbutin levels were observed, fairly similar changes in antibacterial activity occurred (Fig 2B). These results suggest that level of eucalyptol may be an effective measure of antibacterial activity associated with *E*. *globulus*, and that either eucalyptol, or another compound that varies similarly, may be involved in this response. For *T*. *diffusa*, the results suggest that arbutin may be involved in the antibacterial activity associated with this herb, however additional constituents may be involved as well based on the observed differences between Source 1 and Source 2 (Fig 2B).

**Fig 3:**
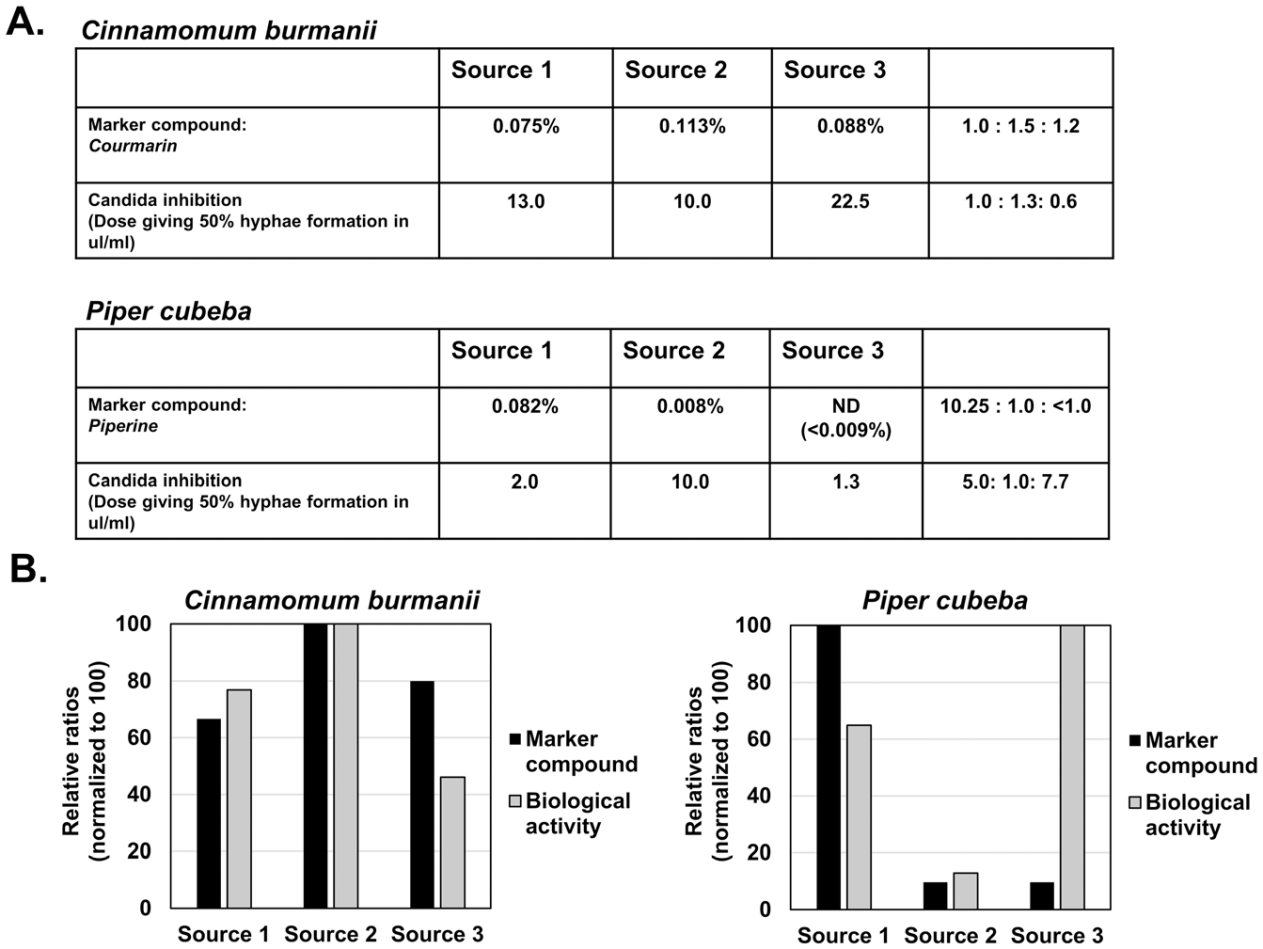
Marker compound level and bioactivity of antifungal herbs. Part A) Marker compound level (percent of total weight) of courmarin (from *C*. *burmanii)* and piperine (from *P*. *cubeba)* and *Candida albcians* hyphal growth ID_50_ values (microliters/ml) are shown from three commercial sources. Part B) Marker compound ratios and bioactivity ratios were normalized to 100 (max. level) and graphed.

The activity and marker compound levels of the antifungal herbs, *Cinnamomum burmanii* and *Piper cubeba* are shown in Fig 3. Our lab has previously demonstrated that these herbs can block the differentiation of *Candida albicans* from a single-celled yeast form to a multicellular hyphal form (data not shown). Bioactivity was measured as the concentration of the extracts required to inhibit *C*. *albicans* hyphal formation by 50% (ID_50_). As shown in Fig 2A, courmarin levels in *C*. *burmanii* varied between 0.075%, 0.113% and 0.088%, whereas piperine levels in *P*. *cubeba* varied between 0.082%, 0.008% and non-detectable (<0.009%). In comparison of the marker compound levels to bioactivity of *C*. *burmanii*, Source 1 and Source 2 agreed well, but Source 3 demonstrated only a weak correlation (Fig 2B). For *P*. *cubeba*, the marker compound level did not correlate with bioactivity, with Source 3 having the lowest piperine level, but the highest bioactivity (Fig 2B). Again, these results suggest that piperine is not a reliable measure of the antifungal activity of *P*. *cubeba* and is likely not involved in the antifungal bioactivity associated with this herb, whereas, for *C*. *burmanii*, courmarin had a weak correlation with bioactivity making it a questionable measure for therapeutic value.

**Fig 4:**
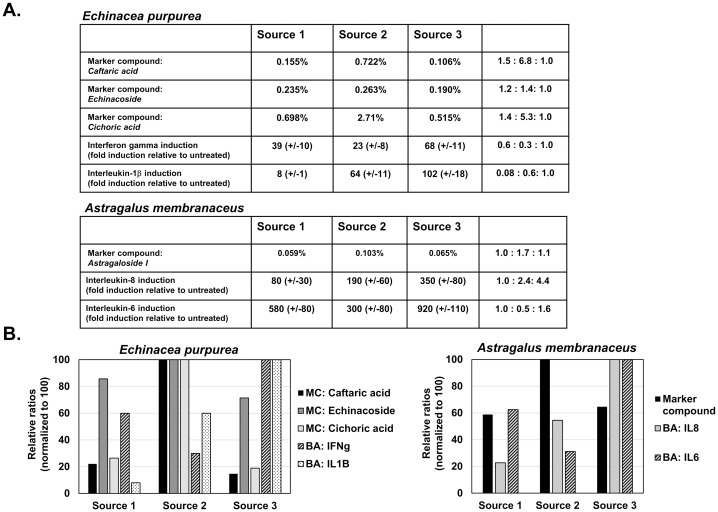
Marker compound level and bioactivity of immune-stimulatory herbs. Part A) Marker compound level (percent of total weight) of cafteric acid, echinacoside and cichoric acid (from *E*. *purpurea)* and astragaloside I (from *A*. *membranaceus)* and cytokine induction values (relative to untreated) are shown from three commercial sources. Part B) Marker compound ratios and bioactivity ratios were normalized to 100 (max. level) and graphed.

Lastly, immune-stimulatory herbs were selected including *Echinacea purpurea* and *Astagulus membranaceus*. For *E*. *purpurea*, three different marker compounds were measured including caftaric acid, echinacoside, and cichoric acid [14]. As shown in Fig 4A, the percentage of all these compounds varied dramatically between the three samples, with the highest levels of all three compounds present in Source 2 and the lowest levels in Source 3. For bioactivity, the ability of the *E*. *purpurea* extracts to induce interferon-gamma (IFN-gamma) and interleukin-1beta (IL1beta) mRNA synthesis in PBMCs was determined. As shown in Fig 4B, the bioactivity measure for either IFNgamma or IL1beta synthesis did not correlate with any of the three marker compounds. This result can most easily been observed in Source 3 which had the highest bioactivity, but lowest marker compound levels (Fig 4B). For *A*. *membranaceus*, the level of astragaloside I varied between 0.059%, 0.103% and 0.065% (Fig 4A). For bioactivity, the ability of *A*. *membranaceus* extracts to induce IL-6 and IL-8 mRNA levels in PBMCs was determined. As shown in Fig 4B, similar results to *E*. *purpurea* were obtained with *A*. *membranaceus* with no clear correlation of bioactivity to marker compound levels.

Initial observation of the results suggests that, for the majority of botanicals tested, a strong correlation did not exist between the marker compound level and the measured bioactivity. Statistical analysis of the samples compared as a whole indicated a significant difference (p < 0.04) supporting that the level of a marker compound is not a consistent indicator of bioactivity. Repeated Measures ANOVA was also used to analyze and compare the three Sources. A statistically significant difference between the three Sources of p < 0.0019 was determined, which may additionally contribute to the disparity between the marker compound level and bioactivity.

**Fig 5:**
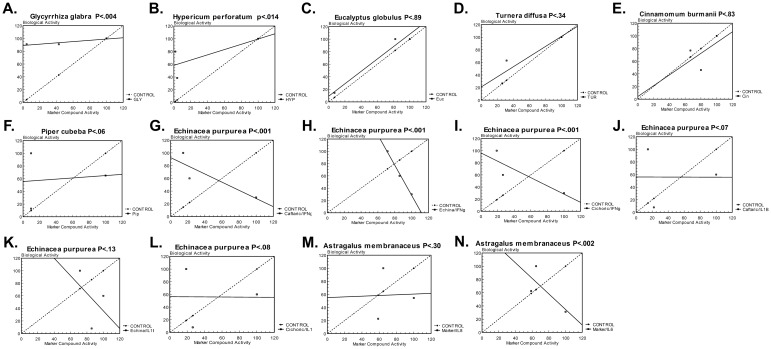
Regression analysis of marker compound level versus bioactivity. A regression line comparison (Co-Variance) between the marker compound level and bioactivity of each botanical is shown. A “CONTROL” regression line representing a 1:1 cause and effect relationship is shown for comparison. The p-value representing the statistical difference between the marker compound level and bioactivity is shown.

Further analysis was required to determine individual group cause and effect relationships between the marker compound level and bioactivity of selected indicators. Therefore, a regression line comparison (Co-Variance) was computed compared to a control regression line (y = 1x + 0) on each of the botanicals, As shown in Fig 5, the control line in each graph represents a perfect 1:1 cause and effect relationship. As supported by Fig 2, *E*. *globulus* demonstrated the strongest cause and effect relationship between the marker compound level and bioactivity (Fig 5C). Similarly, *T*. *diffusa* and *C*. *burmanii* demonstrated a weaker, but possible correlation between the marker compound level and bioactivity (Fig 5D and 5E). For the majority of herbs and all other herbs tested, no cause and effect correlation was observed between the marker compound level and bioactivity, with *G*. *glabra*, *H*. *perforatum*, *E*. *purpurea*, and *A*. *membranaceus* having a significant p < 0.05 supporting a significant difference (Fig 5).

## Discussion

Botanical extracts are composed of numerous constituents or compounds. The amount of each of the specific compounds present in a botanical extract depends on a number of factors including time of harvest, geographical region, agricultural methods, extraction process, and storage methods. For this study, eight different herbs were obtained from three different commercial sources. The initial characterization of these herbs demonstrated substantial variations in levels of the marker compound present in each herb from each of the three sources. As previously noted, the differences observed were likely attributed to environmental variations since all extracts were prepared similarly. When tested for *in vitro* bioactivity the results generally demonstrated a lack of correlation between the variation in marker compound level and the *in vitro* measured bioactivity.

The strongest outlier with possible attributable predicative ability between the marker compound level and bioactivity was *E*. *globulus*. Eucalyptol is the main component in the Eucalyptus essential oil with well-established antibacterial properties [12]. The results showed an overall positive relationship of increased bioactivity as percentage of marker compound increased (Figs 2B and 5C). The established and well documented antibacterial activity of eucalyptol paired with these results suggests the predictive ability of eucalyptol related to the antibacterial bioactivity of *E*. *globulus*. For *T*. *diffusa* and *C*. *burmanii*, a weaker but significant cause and effect relationship was observed between the marker compound level and tested bioactivity. These results suggest that the marker compound measured for these herbs may be involved in the bioactivity, or the activity may involve synergism with additional constituents, or that another, unrelated compound is involved in the bioactivity and follows similar levels as that of the marker compound tested. These unclear correlations with bioactivity make the level of a marker compound questionable as a measure for therapeutic value.

For the majority of botanicals tested, including *G*. *glabra*, *H*. *perforatum*, *P*. *cubeba*, *E*. *purpurea* and *A*. *membranaceus*, the level of the marker compound did not correlate with the tested bioactivity. The concept of botanical standardization using a marker compound is widely accepted as the standard criteria for consistency, quality control, and efficacy of herbal preparations and products [6,7,8,15,16]. The results presented suggest that standardization using a marker compound is not an accurate prediction model for bioactivity. For many herbal products, therapeutic use may be related to the treatment of multiple health conditions. As shown for *E*. *globulus*, if a marker compound like eucalyptol is shown to correlate with bioactivity, standardization using this method may be warranted. However, for the case of *E*. *globulus*, this may only be related to the antibacterial efficacy for this herb. For most botanicals, the specific bioactive constituents have not been identified. Furthermore, phytotherapeutics with multiple therapeutic uses likely contain several different active constituents or contain compounds which work synergistically to produce the desired therapeutic effects. The results presented suggest that standardization of botanical products should be done by an alternative method other than measuring a marker compound which has not been identified as the active constituent. The direct measurement of bioactivity and/or levels of a marker constituent known to be involved in the bioactivity likely offers a more reliable measure of efficacy. However, *in vitro* assays for bioactivity for standardization should be confirmed in *in vivo* model systems as well. In the meantime, until active constituents are identified, *in vitro* bioactivity assays may provide an improved system for evaluating the efficacy and quality assurance in the use of botanical supplements.
